# The Impact of Sodium Alginate Hydrogel on Exogenous Glucose Oxidation Rate and Gastrointestinal Comfort in Well-Trained Runners

**DOI:** 10.3389/fnut.2021.810041

**Published:** 2022-01-20

**Authors:** Shaun Sutehall, Borja Muniz-Pardos, Andrew N. Bosch, Stuart D. Galloway, Yannis Pitsiladis

**Affiliations:** ^1^Division of Physiological Sciences, Department of Human Biology, University of Cape Town, Cape Town, South Africa; ^2^GENUD (Growth, Exercise, Nutrition and Development) Research Group, University of Zaragoza, Zaragoza, Spain; ^3^Faculty of Health Sciences and Sport, University of Stirling, Stirling, United Kingdom; ^4^School of Sport and Health Sciences, University of Brighton, Eastbourne, United Kingdom

**Keywords:** carbohydrate, sodium alginate, running, gastrointestinal distress, carbohydrate oxidation

## Abstract

**Purpose:**

The purpose of this study is to quantify the effect of adding sodium alginate and pectin to a carbohydrate (CHO) beverage on exogenous glucose (ExGluc) oxidation rate compared with an isocaloric CHO beverage.

**Methods:**

Following familiarization, eight well-trained endurance athletes performed four bouts of prolonged running (105 min; 71 ± 4% of VO_2_max) while ingesting 175 mL of one of the experimental beverages every 15 min. In randomized order, participants consumed either 70 g^.^h^−1^ of maltodextrin and fructose (10% CHO; NORM), 70 g^.^h^−1^ of maltodextrin, fructose, sodium alginate, and pectin (10% CHO; ENCAP), 180 g^.^h^−1^ of maltodextrin, fructose, sodium alginate, and pectin (26% CHO; HiENCAP), or water (WAT). All CHO beverages had a maltodextrin:fructose ratio of 1:0.7 and contained 1.5 g^.^L^−1^ of sodium chloride. Total substrate oxidation, ExGluc oxidation rate, blood glucose, blood lactate, serum non-esterified fatty acid (NEFA) concentration, and RPE were measured for every 15 min. Every 30 min participants provided information regarding their gastrointestinal discomfort (GID).

**Results:**

There was no significant difference in peak ExGluc oxidation between NORM and ENCAP (0.63 ± 0.07 and 0.64 ± 0.11 g^.^min^−1^, respectively; *p* > 0.5), both of which were significantly lower than HiENCAP (1.13 ± 0.13 g^.^min^−1^, *p* < 0.01). Both NORM and HiENCAP demonstrated higher total CHO oxidation than WAT from 60 and 75 min, respectively, until the end of exercise, with no differences between CHO trials. During the first 60 min, blood glucose was significantly lower in WAT compared with NORM and HiENCAP, but no differences were found between CHO beverages. Both ENCAP and HiENCAP demonstrated a higher blood glucose concentration from 60–105 min than WAT, and ENCAP was significantly higher than HiENCAP. There were no significant differences in reported GID symptoms between the trials.

**Conclusions:**

At moderate ingestion rates (i.e., 70 g^.^h^−1^), the addition of sodium alginate and pectin did not influence the ExGluc oxidation rate compared with an isocaloric CHO beverage. At very high ingestion rates (i.e., 180 g^.^h^−1^), high rates of ExGluc oxidation were achieved in line with the literature.

## Introduction

It is well established that carbohydrate (CHO) beverage ingestion during prolonged endurance exercise enhances performance (1). This is achieved through several mechanisms such as maintaining blood glucose concentration (2), central effects (3), and attenuating severe dehydration (4). Over the previous ~20 years, efforts have been made to quantify the exogenous CHO (ExCHO) oxidation rates achievable with various combinations and ratios of different CHO sources such as glucose, fructose, and sucrose (5). The maximal ExCHO oxidation rate with the ingestion of glucose is reported as ~1 g^.^min^−1^, even when ingesting glucose at much higher rates (i.e., 108 g^.^h^−1^) (6, 7). To overcome this limitation, it was theorized that by combining multiple CHO sources, a higher ExCHO oxidation rate could be achieved (8). This was evidenced with a significantly higher maximal ExCHO rate (1.75 g^.^min^−1^) elicited following the ingestion of glucose and fructose (154 g^.^h^−1^) in comparison with an isocaloric, glucose beverage (64 g^.^h^−1^) in trained cyclists (8). To achieve this high ExCHO oxidation rate, CHO was ingested at a rate well in excess of the 90 g^.^h^−1^ recommended to athletes performing prolonged endurance events of <2.5 h (9). Whereas this CHO beverage could possibly be considered scientifically “optimal” for maximizing ExCHO oxidation, it is unlikely to be tolerated by many athletes due to the high prevalence of gastrointestinal distress (GID) during prolonged endurance exercise (10, 11), which is often exacerbated with hypertonic CHO beverage ingestion (12).

The precise cause of GID during exercise is not well known, but several mechanisms associated with exercise have been suggested such as residual CHO within the intestine after CHO ingestion (13). The occurrence of GID is higher in running compared with cycling, and it is thought to be caused by the increased body vibration associated with running (14). Although the use of multiple sources of CHO has been reported to reduce the likelihood of GID (15), there are still numerous reports of athletes in “real-life” race scenarios experiencing GID (16). To alleviate these symptoms during competition, these athletes must either reduce exercise intensity or reduce CHO intake, both of which can decrease exercise performance.

Recently, it has been suggested that the addition of sodium alginate and pectin to a CHO beverage will allow a greater concentration of CHO to be ingested without GID (17). It is known that upon contact with the low pH of the gastric fluid, the sodium alginate and pectin will form a pH-sensitive hydrogel which will encapsulate the CHO within the beverage (18). Recent data from our group demonstrate that the addition of a sodium alginate hydrogel enhances the initial gastric emptying (GE) rate of a concentrated CHO beverage at rest, suggesting some impact on intestinal receptors that regulate GE (19). Specifically, the addition of sodium alginate and pectin to a concentrated CHO beverage resulted in a significantly faster GE rate for 20 and 30 min after ingestion compared with and isocaloric control beverage. Despite an enhanced early GE rate at rest, there were no significant differences between the beverages in the concentration of blood metabolites, which may suggest, among other mechanisms, a delayed absorption of CHO from the intestinal lumen due to trapping of CHO within the sodium alginate and pectin hydrogel.

The *in vivo* rate at which CHO is released from the hydrogel into the intestine is not known; however, a pilot study demonstrated that when kept at a constant pH of 6.4 (approximate pH of the duodenum), the gel dissipates in 30 min (19). If the transit time for the hydrogel to travel through the duodenum (site of many glucose-specific transporters) is <30 min, CHO may remain within the hydrogel and released after exiting the duodenum. If this was to occur, the CHO will be more slowly absorbed and may impact the rate of glucose appearance in the blood and subsequent ExCHO oxidation rate. Several studies have investigated ExCHO oxidation of a CHO beverage containing sodium alginate using high CHO ingestion rates, that is, >90 g^.^h^−1^ (20– 23). Notably, with the ingestion of 90 g^.^h^−1^ of maltodextrin and fructose with a ratio of 1:08, ~50 g^.^h^−1^ of glucose is ingested, approaching the maximum intestinal absorption rate of glucose (6). In this instance, it may not be possible to observe small differences in the absorption and subsequent oxidation rate of ingested glucose. To test the potentially delayed absorption of CHO when encapsulated within a sodium alginate and pectin hydrogel, a more moderate ingestion rate of maltodextrin (i.e., ~35 g.h^−1^) was used to ensure that the glucose-specific transports are not saturated and glucose is rapidly absorbed from the intestine. One study has investigated the use of sodium alginate and pectin when ingesting CHO at 70 g^.^h^−1^ (24) but did not measure the ExCHO oxidation rate, and thus, further research is required to draw conclusions. Therefore, the aim of this study was, first, to investigate whether adding sodium alginate and pectin to a CHO beverage decreased the ExCHO oxidation rate during prolonged, steady-state running compared with an isocaloric non-encapsulated CHO at moderate ingestion rates. Second, to determine whether a high ExCHO can be achieved with the ingestion of a highly concentrated CHO beverage containing sodium alginate and pectin.

## Methods

### Participants

Fourteen male, well-trained endurance athletes were recruited for this study from University and local running clubs. Six participants withdrew from the study either due to an injury unrelated to the study (*n* = 3) or were unable to continue with the time commitment required of the study (*n* = 3). Therefore, eight well-trained athletes remained and completed all trials (age: 28 ± 9 years, height: 178 ± 7 cm, body mass: 69.0 ± 8.5 kg, maximal oxygen consumption [VO_2_max]: 69.9 ± 8.1 mL^.^kg^.^min^−1^) with median (range) personal best times of 15:48 (14:45–18:39) min:sec and 34:00 (32:10–43:00) min:sec for 5 and 10 km, respectively. Each participant was provided with written details of the study methods and had them verbally explained before written informed consent was signed and collected. This study was approved by the Ethics Committee of the University of Stirling prior to participant recruitment.

### Preliminary Testing and Familiarization

One week prior to the familiarization trial, all participants performed an incremental running test to determine VO_2_max which was performed on a motorized treadmill (Pulsar 3p, h/p/cosmos, Nussdorf, Germany). Upon arrival, participants' near nude body mass and height were recorded. Following a 10–15-min self-selected warm-up, participants began running at 9 km^.^h^−1^ for one min, followed by increments of 1 km^.^h^−1.^min^−1^ until 17 km^.^h^−1^ was reached; thereafter, the speed remained constant, and the gradient increased by 1%^.^min^−1^ until volitional exhaustion. Breath by breath measurements were recorded continuously throughout the exercise duration *via* a metabolic analyzer (Quark CPET, COSMED, Rome, Italy) which was calibrated prior to each test. Heart rate was recorded continuously *via* radiotelemetry heart rate monitor (Forerunner 210, Garmin, Kansas, USA). The VO_2_ values of the last 15 s of each stage were averaged, with the highest VO_2_ value obtained considered as VO_2_max. After a minimum of 7 days had elapsed following the VO_2_max trial, all participants performed a familiarization trial which emulated the exercise trial detailed below while drinking distilled water at the same ingestion rates as the main trials. From the VO_2_max trial, the ventilatory threshold was visually determined as the point at which the increase in ventilation (VE) became disproportional to the increase in VO_2_. The initial treadmill running speed was set at 80% of ventilatory threshold and at a 1% gradient with the speed adjusted according to the participants feedback to elicit a speed for the 105 min run that would be considered as “hard” by participants (i.e., ~15 RPE) during the familiarization trial. This approach was taken to ensure that participants could complete the 105 min run while maintaining a steady-state metabolic response, which was required for valid estimation of substrate metabolism from expired gases.

### Diet and Activity Prior to Exercise Trials

All participants were asked to record a food and activity diary 24 h prior to attending the familiarization trial and to maintain the same diet and activity before each subsequent trial. Participants were also asked to perform a 60-min “glycogen depleting” exercise bout within 5 days of the experimental trials to greatly reduce the stores of ^13^C in the body. To prevent the consumption of ^13^C enriched foods, all participants were provided a list of foods to avoid (specifically CHO derived from C_4_ plants, e.g., corn and sugar cane) and asked to refrain from these foods over the entire duration of the study period.

### Exercise Protocol

Participants reported to the laboratory in the morning (7:00 am or 10:30 am) after an overnight fast and having abstained from caffeine, alcohol, and strenuous exercise for the previous 24 h. Each participant arrived at the laboratory the same time for each trial to avoid any influence of the circadian rhythm. Upon arrival at the laboratory, participants voided their bladder and near nude body mass was measured. Subsequently, a flexible, 21-gauge cannula (Nexiva, Becton Dickinson, Devon, UK) was inserted into an antecubital vein and attached to an extension tubing. The cannula was kept patent by flushing with 5 mL of 0.9% isotonic saline after each blood sample collection.

The participant performed a 10- to 15-min warm-up at a self-selected speed on a motorized treadmill, and upon completion, participants were allowed ~5 min of rest to stretch, if required. The participant then mounted the treadmill, and a 6 mL of resting blood sample was taken. Once this was completed, 175 mL of the experimental drink was consumed, and the participant began to run at the speed determined during the familiarization test for 105 min, with a 1% gradient set during all trials. For every 15 min during the trial, breath by breath VO_2_, carbon dioxide production (VCO_2_), and respiratory exchange ratio (RER) were measured *via* a metabolic analyzer for 5 min with the values collected during the final 2.5 min averaged and reported. Immediately after this, expired end-tidal breath samples were collected into a 750-mL bag, with the initial 400 mL of the breath removed through an additional discard bag. Duplicate samples of expired end-tidal breath were drawn into a syringe which was then injected into a 10-mL Exetainer tube without contact with room air (Labco Ltd, High Wycombe, UK) and a 6 mL of blood sample was taken. Throughout every trial, a standing fan was used to cool the participants, reducing the thermal strain. The average ambient temperature and relative humidity during the trials were 19 ± 2°C and 43 ± 7%, respectively. The average running intensity maintained during the trials was 71 ± 4% VO_2_max.

### Experimental Beverages

The beverages consumed during the trials were either a 10% maltodextrin and fructose drink (NORM, osmolality: 374 ± 14 mOsm^.^kg^−1^), a 10% maltodextrin and fructose drink with an additional 0.2% sodium alginate and pectin (ENCAP, osmolality: 377 ± 11 mOsm^.^kg^−1^), both providing 70 g^.^h^−1^, or distilled water (WAT). A third beverage was included to determine whether the addition of sodium alginate and pectin would permit very high concentrations of CHO to be tolerated and to assess whether the hydrogel will inhibit maximal ExCHO oxidation rates. This drink was a 26% maltodextrin and fructose drink with an additional 0.2% sodium alginate and pectin (HiENCAP, osmolality: 1,158 ± 72 mOsm^.^kg^−1^), providing 180 g^.^h^−1^. All CHO beverages contained an additional 1.5 g^.^L^−1^ NaCl, had a maltodextrin:fructose ratio of 1:0.7, and were ingested at a rate of 700 mL^.^hr^−1^. The order of all drinks was randomly allocated according to a Latin square crossover design and provided in a single-blind manner. Each trial was separated by a minimum of 7 days.

### Subjective Measures

The participants were asked to report their rating of perceived exertion (RPE) on the 6 to 20 Borg category scale (25) every 15 min and were asked to rate various GI symptoms on a 0 to 9 scale for every 30 min, based on a previously validated questionnaire (26). The participants were asked verbally for each symptom and responded with their current sensation on a 0–9 scale (0 = none at all, 9 = the worst ever). The symptoms related to the upper gastrointestinal tract included were reflux or heartburn, belching, bloating, stomach pain or cramps, vomiting, and nausea. The symptoms related to the lower gastrointestinal tract included were lower abdominal cramps, flatulence, urge to defecate, and diarrhea. When a score of five or above was given, it was considered as severe and would likely impact upon exercise performance as this is the point at which symptoms progress from “moderate” (rating of four) to “serious” (rating of five). A summative value per symptom for each beverage was calculated by summing every individual score at every time point and combining participants' scores (maximum value per participant = 36 and maximum value per trial = 252).

### Blood and Breath Analysis

At every 15-min time point, blood samples were collected into a 6-mL serum tube and 100 μL of whole blood placed directly into 1 mL of ice cold 0.4 M perchloric acid, vigorously mixed, and after the completion of the trial stored at −20°C until analysis of blood lactate concentration. After the completion of the trial, blood samples collected within the serum tubes were centrifuged at 2,000 g for 10 min, while held at a temperature of 4°C. The resulting serum was aliquoted into 1.5-mL microcentrifuge tubes and stored at −20°C until analysis of glucose and non-esterified fatty acid (NEFA). Glucose, lactate, and NEFAs were analyzed using enzymatic assay methods on an automated analyzer (iLab Aries, Instrumentation Laboratory, Warrington, UK), with a between-assay coefficient of variation of 2.5%. Urine osmolality was measured *via* freezing point depression prior to sample freezing (Type 15 Osmometer; Löser Messtechnik, Berlin, Germany).

Breath samples were analyzed for ^13^C:^12^C ratio using a GC-IRMS (Europa Scientific, Crew, UK). Each sample was flushed into a packed column gas chromatograph, which held at 60°C, and the resulting chromatographic peak passed into the GC-IRMS where isotopomers at 44, 45, and 46 m/z for CO_2_ were measured, from which the ^13^C value is determined.

### Calculations for Substrate Oxidation

Total CHO and fat oxidation rates (g^.^min^−1^) were calculated using VO_2_ and VCO_2_ (L^.^min^−1^) using stoichiometric equations (27), and assuming protein oxidation during exercise was negligible:


(1) $$\text{CHO oxidation }({\text{g}}^{.}{\text{min}}^{-\text{1}})\text{ = 4}.\text{210 }{\text{VCO}}_{2}\text{ - 2}.\text{962 }{\text{VO}}_{2}$$

(2) $$\text{Fat oxidation }({\text{g}}^{.}{\text{min}}^{-\text{1}})\text{ = 1}.\text{695 }{\text{VO}}_{2}\text{ - 1}.\text{701 }{\text{VCO}}_{2}$$

To quantify the ExCHO oxidation rate of these drinks, the maltodextrin and fructose used were from naturally enriched sources and thus contained a high abundance of ^13^C. The natural enrichment for NORM, ENCAP, and HiENCAP was −11.71, −11.92, and −11.77%, respectively. To further increase the abundance of ^13^C, 50 mg^.^L^−1^ of D-glucose-^13^C_6_ tracer was added to each CHO drink. The resulting ^13^C enrichments for NORM, ENCAP, and HiENCAP were 28.1, 26.9, and 4.0%, respectively. Due to the addition of 50 mg^.^L^−1^ of D-glucose-^13^C_6_ tracer within each beverage, the CHO was not equally labeled and therefore, ExCHO oxidation could not be estimated accurately. As ExCHO could not be determined, exogenous glucose (ExGluc) oxidation was calculated by estimating the total amount of ^13^C consumed by the participant that is derived from the glucose within the beverage (i.e., enriched maltodextrin and D-glucose-^13^C_6_ tracer) and adjusting the drink enrichment to account for the “excess” ^13^C derived from the enriched fructose. The equations of Young et al. were used (28), incorporating equations by Peronnet et al. (29). All statistical comparisons of ExGluc oxidation rate were performed after 60 min of running in order for the release of ^13^CO_2_ from the body's bicarbonate pool to stabilize and reflect oxidation rate. The ^13^C enrichment of the expired breath samples was expressed as the difference between the ^13^C:^12^C ratio of the sample and the international standard, Pee Dee Belemnite (PDB), and ExGluc oxidation calculated using the following equation:


(3) $$\begin{array}{l} \text{ExGluc oxidation }({\text{g}}^{.}{\text{min}}^{\text{–1}})\\ =\;VCO_{2}[(R_{\text{exp}}\;-\;R_{\text{ref}})/(R_{\text{exo}}-R_{\text{ref}})]/k \end{array}$$

where VCO_2_ is expressed in L^.^min^−1^, R_exp_ is the isotopic composition of the expired CO_2_, R_ref_ is the isotopic composition of expired CO_2_ during the WAT trial, R_exo_ is the isotopic composition of the ingested CHO within the drink, and k is the volume of CO_2_ produced through the complete oxidation of glucose (0.747 L^.^g^−1^). By subtracting the ExGluc oxidation rate from total CHO, “other CHO” can be estimated which includes both endogenous CHO oxidation (i.e., liver and muscle glycogen) and exogenous fructose oxidation. Since comparison of ExGluc oxidation rate can only be performed after 60 min, the calculation of relative contribution to energy expenditure includes data from 60–105 min only.

### Statistical Analysis

All data are presented as mean ± standard deviation (SD). All data were assessed for normality using a Shapiro–Wilk test and violations of sphericity corrected using Greenhouse–Geisser epsilon. A two-way analysis of variance (ANOVA) with repeated measures (time^*^drink) was used to identify interactions in substrate oxidation. In the case of non-normally distributed data (i.e., RPE and GID symptoms), a Friedman test was performed and if significant, followed by a Wilcoxon signed-rank test. Differences in blood and serum metabolites, peak ExGluc oxidation rate, urine volume, urine osmolality, and body mass between experimental conditions were assessed using a one-way ANOVA and where significance was found, which was followed by Tukey's *post hoc* for multiple comparisons. Differences within a condition (i.e., pre vs. post trial) were assessed using a paired sample *t*-test. Due to the high number of dropouts, a *post hoc* order effect analysis was performed, which showed no significant effect of trial order on physiological variables. Statistical analysis was performed using GraphPad (version 8.0, GraphPad Software Inc, CA, USA) and SPSS (version 25.0, IBM Corp, NY, USA). Data were declared as significant if *p* ≤ 0.05.

## Results

There were no significant differences in body mass between any of the trials prior (NORM: 69.0 ± 10.0, HiENCAP: 69.0 ± 10.0, WAT 69.0 ± 9.8, ENCAP: 68.7 ± 9.8 kg) or after the 105-min run (NORM: 67.9 ± 10.1, HiENCAP: 68.0 ± 9.9, WAT 68.0 ± 9.7, ENCAP: 67.7 ± 9.7 kg). Body mass significantly decreased within each experimental trial (*p* < 0.01), with a mean reduction of 1.5%.

### RER and Substrate Oxidation

**Figure 1:**
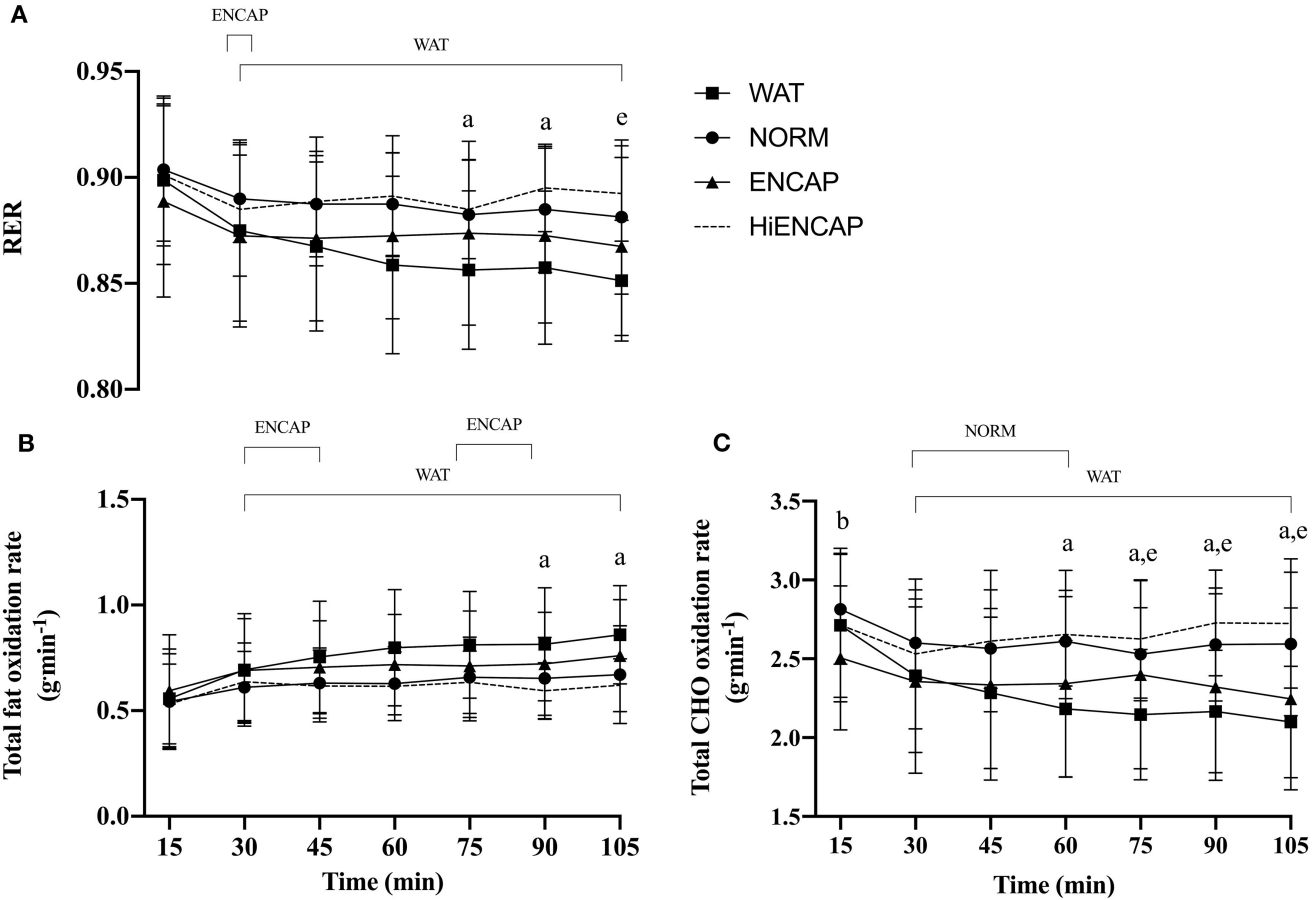
RER **(A)**, total fat oxidation rate **(B)**, and total CHO oxidation rate **(C)** over the 105-min run between HiENCAP (dashed gray line), NORM (filled circles), ENCAP (filled triangles), and WAT (filled squares). a = significant difference between NORM and WAT, b = significant difference between NORM and ENCAP, and e = significant difference between WAT and HiENCAP. Time points that are significantly different from values at 15 min are encompassed by a square bracket. *n* = 8. Values are mean ± SD.

There was a significant effect of time for HR, VO_2_, and VE (*p* < 0.05, Table 1), but there was no main effect of drink or an interaction effect between trials. Whereas there was no main effect of drink on RER, there was a significant main effect of time (*p* < 0.01, Figure 1A) in addition to an interaction effect. There were no significant differences between any of the experimental drinks in RER between 15 min and 60 min. Thereafter, both NORM and HiENCAP were significantly higher than WAT.

**Table 1:** Cardiorespiratory variables collected during a 105 min run at 71 ± 4% VO_2_max.

	**Time (min)**						
	**15**	**30**	**45**	**60**	**75**	**90**	**105**
**HR (beats** ^**.**^ **min** ^**−1**^ **)**							
WAT	151 ± 9	155 ± 8^*^	155 ± 8^*^	156 ± 10^*^	157 ± 13	159 ± 9^*^	160 ± 11^*^
NORM	152 ± 9	153 ± 12	153 ± 12	154 ± 12	155 ± 12	156 ± 12	158 ± 11
ENCAP	151 ± 10	156 ± 12	157 ± 10^*^	156 ± 11	158 ± 10^*^	159 ± 12^*^	160 ± 12^*^
HiENCAP	152 ± 9	155 ± 8^*^	156 ± 9^*^	156 ± 10	158 ± 10	158 ± 11	160 ± 11
**VO**_**2**_ **(L**^**.**^**min**^**−1**^**)**							
WAT	3.32 ± 0.26	3.33 ± 0.29	3.37 ± 0.30	3.38 ± 0.31	3.37 ± 0.31	3.39 ± 0.34	3.43 ± 0.35
NORM	3.37 ± 0.33	3.34 ± 0.33	3.35 ± 0.28	3.38 ± 0.31	3.38 ± 0.30	3.42 ± 0.31	3.45 ± 0.32
ENCAP	3.17 ± 0.25	3.30 ± 0.25	3.33 ± 0.26	3.37 ± 0.27	3.41 ± 0.24^*^	3.39.23^*^	3.43 ± 0.25^*^
HiENCAP	3.28 ± 0.29	3.34 ± 0.27	3.36 ± 0.26	3.39 ± 0.24	3.40 ± 0.25^*^	3.41 ± 0.25^*^	3.46 ± 0.24^*^
**VE (L** ^**.**^ **min** ^**−1**^ **)**							
WAT	85.1 ± 7.7	86.2 ± 9.2	87.0 ± 8.0	88.3 ± 8.6	88.6 ± 8.9	90.1 ± 9.3	91.8 ± 11
NORM	86.8 ± 10.2	85.3 ± 8.5	86.7 ± 8.8	89.4 ± 9.9	90.3 ± 9.9	91.2 ± 9.4	93.4 ± 10.4
ENCAP	80.0 ± 9.0	84.9 ± 8.5	86.5 ± 10.2	87.6 ± 9.7	90.0 ± 9.2^*^	89.4 ± 9.9^*^	91.1 ± 10.3^*^
HiENCAP	85.1 ± 11	84.7 ± 10.3	86.3 ± 10.8	89.2 ± 9.3	89.9 ± 9.1	91.6 ± 9.5^*^	93.4 ± 10.2^*^
**RPE**							
WAT	11 ± 1	11 ± 2	12 ± 2	13 ± 1	13 ± 1	14 ± 1	15 ± 2
NORM	11 ± 2	12 ± 2	12 ± 1	13 ± 1	13 ± 1	14 ± 1	14 ± 1
ENCAP	11 ± 1	12 ± 1	12 ± 1	13 ± 1	13 ± 1	13 ± 1	14 ± 1
HiENCAP	11 ± 2	11 ± 2	12 ± 1	13 ± 1	13 ± 1	14 ± 1	14 ± 1

*There were no significant differences between the ingestion of WAT (water), NORM (70 g^.^hr^−1^ maltodextrin and fructose), ENCAP (70 g^.^h^−1^ maltodextrin, fructose, sodium alginate and pectin) or HiENCAP (180 g^.^h^−1^ maltodextrin, fructose, sodium alginate and pectin) on heart rate (HR), oxygen consumption (VO_2_), ventilation rate (VE) or rating of perceived exertion (RPE)*.

* *= significantly different from 15 min time point*.

There was no main effect of drink on the rate of fat oxidation between trials, but there was a significant main effect of time (*p* < 0.01, Figure 1B) and a time^*^drink interaction (*p* < 0.01). There were no significant differences in the rate of fat oxidation between the experimental drinks until 75 min, after which NORM was significantly lower than WAT.

There were significant main effects for drink and time in addition to a time^*^drink interaction (*p* < 0.01) on estimated whole-body CHO oxidation rate (Figure 1C). From 60 min until the competition of the exercise, NORM had a significantly higher estimated whole-body CHO oxidation rate than WAT. Similarly, HiENCAP was significantly higher than WAT from 75 min until the completion of the 105 min.

**Figure 2:**
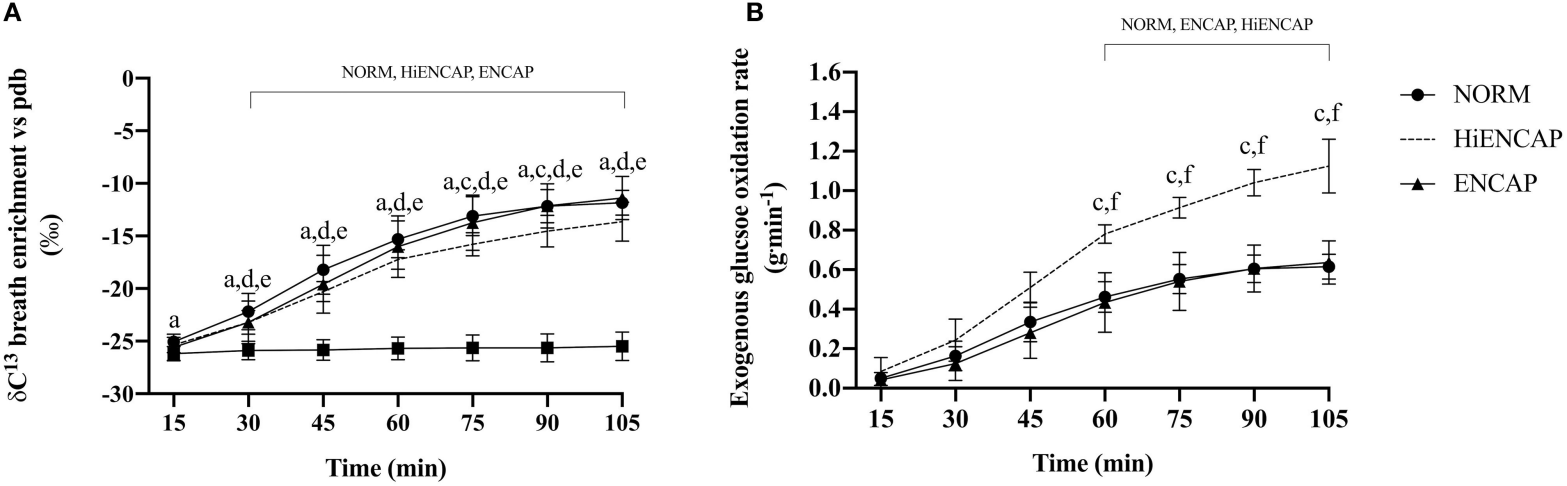
Breath ^13^CO_2_ enrichment vs. PDB **(A)** and ExGluc oxidation **(B)**. a = significant difference between NORM and WAT, c = significant difference between NORM and HiENCAP, d = significant difference between WAT and ENCAP, e = significant difference between WAT and HiENCAP, and f = significant difference between ENCAP and HiENCAP. Time points that are significantly different from values at 15 min are encompassed by a square bracket. *n* = 8. Values are mean ± SD.

There was a significant main effect for drink (*p* < 0.01) and time (*p* < 0.01) in addition to a time^*^drink interaction (*p* < 0.01) for δ^13^CO_2_ in expired breath (Figure 2A). From 30 min onward, each CHO beverage had significantly higher breath ^13^CO_2_ enrichment compared with WAT. NORM demonstrated a significantly higher ^13^CO_2_ enrichment compared with HiENCAP at 75 and 90 min. There were no other significant differences between any of the CHO trials.

There were significant main effects for both drink (*p* < 0.01) and time (*p* < 0.01) in addition to a time^*^drink interaction (*p* < 0.01) for ExGluc oxidation rate (Figure 2B). There were no significant differences in ExGluc oxidation rates between NORM and ENCAP from 60 to 105 min. Peak ExGluc oxidation rate was significantly higher with HiENCAP (1.13 ± 0.13 g^.^min^−1^) than ENCAP (0.64 ± 0.11, *p* < 0.01) and NORM (0.63 ± 0.07, *p* < 0.01) with no differences between NORM and ENCAP.

**Figure 3:**
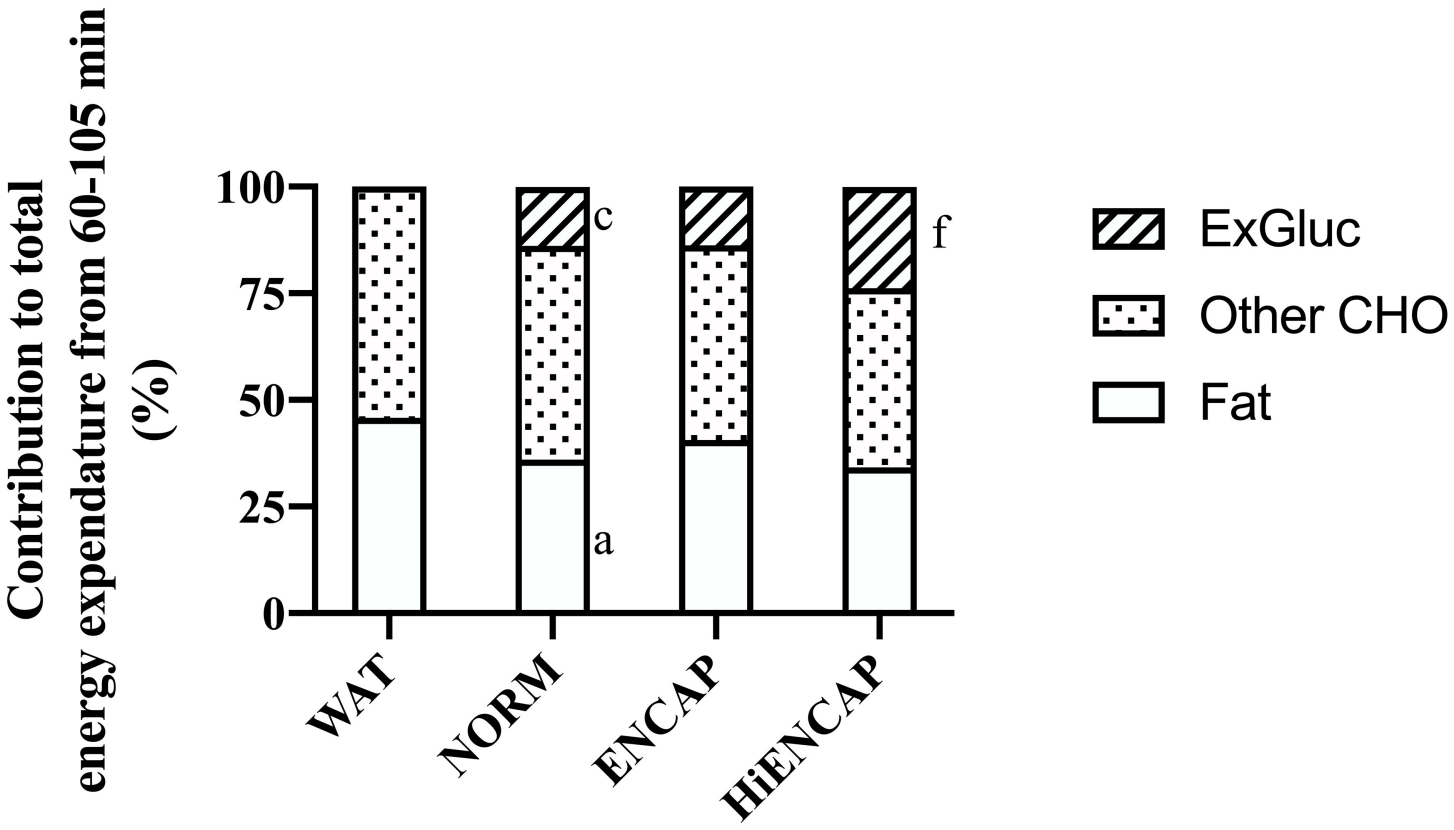
Relative contribution to energy expenditure from 60–105 min. a = significant difference between NORM and WAT, c = significant difference between NORM and HiENCAP, and f = significant difference between ENCAP and HiENCAP. *n* = 8. Values are mean.

The contribution of ExGluc to total energy expenditure was significantly higher with the ingestion of HiENCAP (24 ± 2%) than both NORM (14 ± 2%, *p* < 0.01) and ENCAP (14 ± 3%, *p* < 0.01) with no difference between NORM and ENCAP (Figure 3). There was no significant difference in the contribution of other CHO to total energy expenditure between any of the trials. From 60 to 105 min, the contribution of fat to total energy expenditure with NORM was significantly less than WAT (36 ± 9 vs. 46 ± 12%, respectively) with no other differences between trials.

### Blood Metabolites and Urine

**Figure 4:**
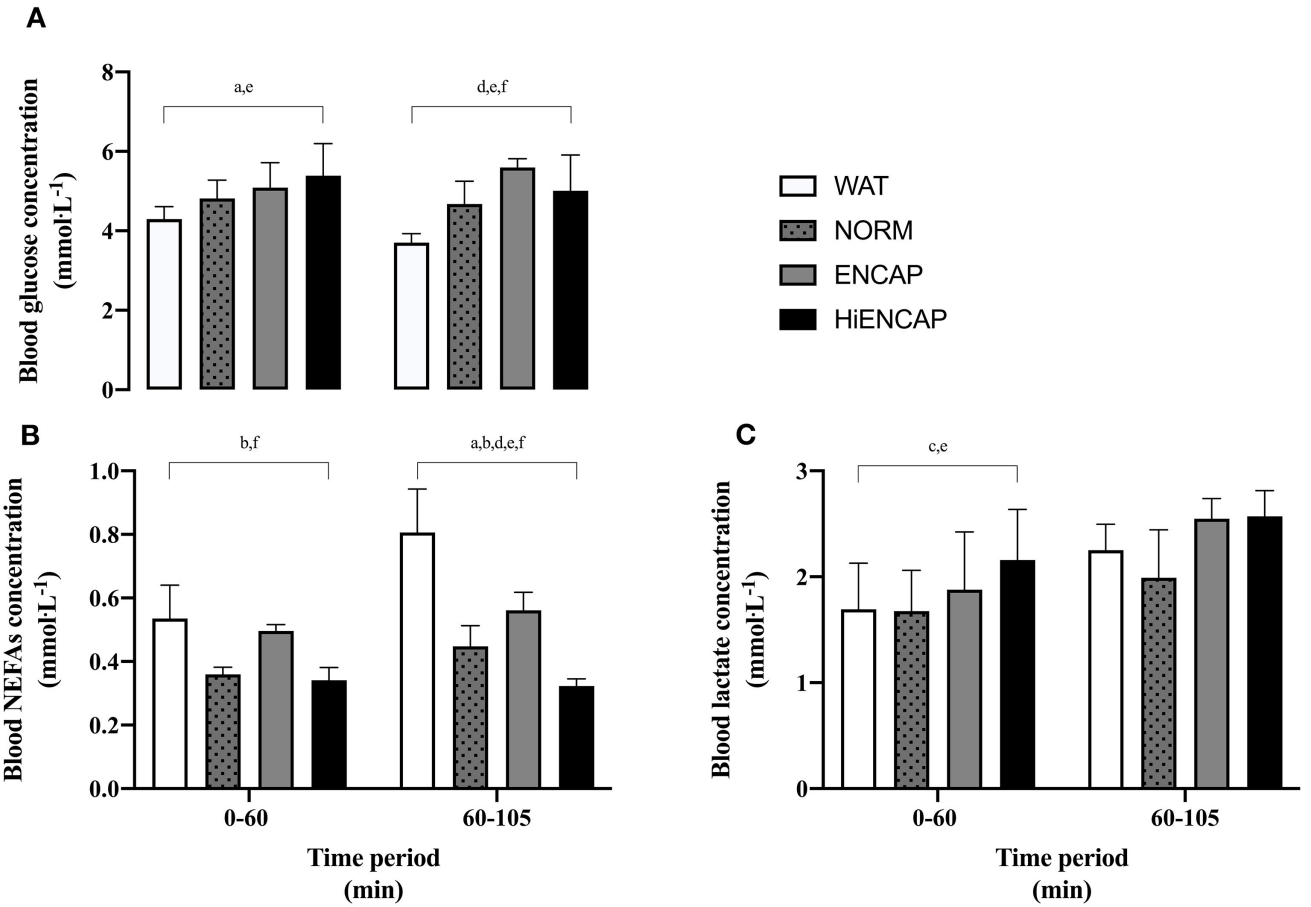
Blood glucose **(A)**, serum NEFAs **(B)**, and blood lactate **(C)** over the 105-min run between WAT (white), NORM (dotted gray), ENCAP (gray), and HiENCAP (black). a = significant difference between NORM and WAT, b = significant difference between NORM and ENCAP, c = significant difference between NORM and HiENCAP, d = significant difference between WAT and ENCAP, e = significant difference between WAT and HiENCAP, and f = significant difference between ENCAP and HiENCAP. Blood glucose *n* = 5 and serum NEFAs and blood lactate *n* = 7. Values are mean ± SD.

Blood glucose, NEFAs, and blood lactate concentrations are shown in Figure 4. Due to issues with cannulation, blood could not be drawn from one participant, and additionally, incomplete data were collected for two participants and subsequently removed from analysis (blood glucose; *n* = 5, serum NEFAs; *n* = 7, and blood lactate; *n* = 7). Due to low participant number and therefore a reduced statistical power, data are presented as the mean response for each trial from 0 to 60 min and from 60 to 105 min.

From 0 to 60 min, there were significant differences in blood glucose concentration between both NORM and HiENCAP when compared to WAT (Figure 4A). From 60 to 105 min, glucose concentration was significantly higher in ENCAP than HiENCAP, both of which were significantly higher than WAT. Over the initial 0–60 min, serum NEFAs were significantly lower in NORM and HiENCAP compared with ENCAP (Figure 4B). From 60 to 105 min, serum NEFAs with WAT were significantly higher than each CHO beverage and higher in ENCAP than both NORM and HiENCAP. HiENCAP demonstrated a significantly higher blood lactate concentration than NORM and WAT (Figure 4C) during the first 60 min, but there were no significant differences between the beverages from 60 to 105 min.

There were no significant differences in urine osmolality before or after exercise between the experimental drinks. There were no differences in the urine osmolality when comparing pre and post trial urine osmolality within each experimental condition, with the exception with NORM, which significantly decreased after the trial (745 ± 249 vs. 539 ± 328 mOsmol^.^kg^−1^, respectively). There were no significant differences in the volume of urine produced between any of the experimental drinks.

### Subjective Response

There was a significant main effect of time on RPE, but there was no main effect of beverage or an interaction effect (Table 1). The RPE in all trials began at 11 after 15 min and rose to 14 at 105 min with the exception of WAT, which ended with an RPE of 15. Subjective measures of GID are presented in Table 2. There was no significant difference in any GID symptom between the different drinks; however, considering the relatively small number of participants, the use of non-parametric statistics, and the comparisons between multiple treatments, we also present the GID results in a qualitative manner. There was no incidence of “severe” ratings for all eight upper or lower GID for NORM and ENCAP (24 total symptom ratings) and only 4% (one incidence) of a severe rating within water, specifically, one participant reported a rating of seven for flatulence after 45 min of running. In HiENCAP, there was a larger rate of severe incidences of GID, specifically, belching (7%), bloating (14%), flatulence (11%), and urge to defecate (14%). Similarly, the highest absolute cumulative scores for any GID symptom were achieved with the ingestion of HiENCAP.

**Table 2:** Values are overall summative accumulation scores for each trial of all those participants who reported a symptom (*n* = 7), and the range of summative scores for individual participants is shown in parentheses.

	**WAT**		**NORM**		**ENCAP**		**HiENCAP**	
	**Incidence**	**GID**	**Incidence**	**GID**	**Incidence**	**GID**	**Incidence**	**GID**
	**(severe)**	**rating**	**(severe)**	**rating**	**(severe)**	**rating**	**(severe)**	**rating**
**Upper symptoms**								
Reflux/heartburn	0%	5 (1–2)	0%	7 (3–4)	0%	3 (3–3)	0%	4 (4–4)
Belching	0%	11 (1–4)	0%	23 (2–10)	0%	8 (1–4)	7%	34 (1–13)
Bloating	0%	29 (2–13)	0%	16 (1–6)	0%	25 (1–9)	14%	43 (2–24)
Stomach pain	0%	8 (1–4)	0%	16 (2–8)	0%	11 (1–4)	0%	12 (1–5)
Urge to regurgitate	0%	3 (3–3)	0%	4 (1–3)	0%	6 (2–4)	0%	9 (1–3)
**Lower symptoms**								
Lower abdominal cramp	0%	9 (3–6)	0%	8 (1–4)	0%	10 (2–4)	0%	5 (2–3)
Flatulence	4%	37 (2–10)	0%	28 (3–8)	0%	20 (2–7)	11%	43 (1–22)
Urge to defecate	0%	24 (3–13)	0%	24 (1–12)	0%	31 (5–10)	14%	52 (1–23)
**Others**								
Nausea	0%	6 (3–3)	0%	8 (3–5)	0%	5 (1–4)	0%	5 (1–3)
Dizziness	0%	10 (1–5)	0%	12 (2–7)	0%	10 (1–4)	0%	11 (2–5)
Headache	0%	12 (2–6)	0%	9 (2–4)	0%	4 (4–4)	0%	10 (2–4)
Side ache/stitch	0%	6 (1–5)	0%	9 (4–5)	0%	6 (2–4)	0%	10 (1–4)
Muscle cramp	7%	30 (1–18)	11%	43 (1–16)	11%	23 (4–17)	7%	25 (2–18)

*A summative value per symptom for each beverage was calculated by summing every individual score at every timepoint and combining participants' scores (maximum value per participant = 36 and maximum value per beverage = 252). The incidence of severe GID for each symptom was calculated as the% of all ratings for a beverage that was reported as severe (i.e., ≥5). There were no significant differences between trials*.

## Discussion

In line with the recently published literature, the addition of sodium alginate and pectin did not alter the rate of ExGluc oxidation when compared to an isoenergetic CHO beverage. HiENCAP elicited a ~56% higher peak ExGluc oxidation rate than NORM and ENCAP, and values were similar to the highest ExGluc oxidation rates reported in the literature (30). Direct comparisons with the literature cannot be made as no isocaloric control for HiENCAP was included in this study. Whole-body CHO oxidation with ENCAP was not significantly different from either WAT or NORM throughout the entirety of the exercise duration. There were no differences in the occurrence of GID among NORM, ENCAP, and HiENCAP.

The addition of sodium alginate and pectin to a CHO beverage has received considerable attention from the academic literature (17, 19, 20, 23, 24, 31–33) and the general media (34). The proposed beneficial mechanism of adding sodium alginate and pectin to a CHO beverage relies on the encapsulation of CHO within a pH-sensitive hydrogel in the stomach, which is transported into the intestine, where the increase in pH will dissipate the hydrogel, releasing the CHO for absorption (17). The time course of this release is dependent on the hydrogel formulation, which can be tailored to release its contents at a desired rate (35). Recently, we demonstrated that the early GE rate of a single, large bolus of a concentrated CHO beverage is enhanced with the addition of sodium alginate and pectin at rest (19). This finding suggests that the hydrogel trapping CHO has an impact on the intestinal CHO or osmoreceptors and thus attenuates the inhibition of GE typically associated with concentrated CHO solution emptying. Despite the enhanced early GE rate, circulating blood metabolites were not impacted. If intestinal absorption was reduced due to delayed hydrogel dissipation, it would likely slow substrate delivery thereby affecting ExCHO oxidation. However, in this study, we observed that encapsulating the CHO within a hydrogel had no effect on ExGluc oxidation rate, at ingestion rates unlikely to saturate intestinal transporters (i.e., ~42 g^.^h^−1^ of glucose). Considering that the saturation point of SGLT1 has been suggested to be ~1.0–1.2 g^.^min^−1^ (5, 30), the ingestion rate of maltodextrin used within this study (i.e., 0.7 g^.^min^−1^) is below maximal absorption rates. Since intestinal absorption of CHO at this ingestion rate is not expected to be a limiting factor for ExGluc oxidation, any differences related to the action of the hydrogel on intestinal absorption would affect the ExGluc oxidation rate in ENCAP. It must be acknowledged that these proposed actions relating to a faster early GE and theoretical slower intestinal absorption are based on observations over the first ~30 min after the ingestion of a single bolus of CHO containing sodium alginate and pectin (19) while in a seated position. Therefore, this finding may not extrapolate after the initial 20–30 min during exercise or with repeated ingestion of a CHO beverage containing sodium alginate and pectin.

During the initial 60 min of exercise, blood glucose concentration was higher with the ingestion of NORM and HiENCAP compared with WAT (Figure 4A). Despite a larger mean difference in blood glucose concentration than between NORM and WAT, ENCAP did not differ from WAT significantly. This is likely due to the larger data variability with ENCAP; a trend that was also observed in the GE rate of an ingested beverage containing sodium alginate and pectin (19), which was potentially caused by larger individual differences in rates of hydrogel formation or dissolution with ENCAP. Through the entire 105 min, ENCAP demonstrated a significantly higher concentration of serum NEFAs than both NORM and HiENCAP which may be indicative of an increased mobilization and utilization of fatty acids for metabolism, something that has been highlighted by others (20, 32). The cause of this is unknown and further research should explore any potential mechanisms of the hydrogel formation or dissolution on fat oxidation. Due to the low sample size analyzed for blood metabolites within this study, these data should be interpreted with caution.

Since the first study discussing the potential benefits of adding sodium alginate and pectin to a CHO beverage (17), there has been a growing interest in exploring and quantifying any ergogenic or ergolytic effects (18, 20–24). Recently, a study by McCubbin et al. investigated the effects of sodium alginate and pectin when ingesting 1.5 g^.^min^−1^ of CHO during prolonged cycling (180 min) at 60% VO_2_max compared with a CHO and sodium-matched beverage (31). The main findings of this study (31) revealed that there were no significant differences in GID, CHO malabsorption, blood glucose concentration, total CHO, and fat oxidation or performance during an incremental exercise to exhaustion. Similarly, Baur et al. showed no differences in substrate oxidation, blood metabolites, repeated sprint performance or GID between a CHO beverage containing sodium alginate and pectin, or an isocaloric CHO beverage, which provided at 1.3 g^.^min^−1^ (32). Another study compared the ingestion of 1.5 g^.^min^−1^ of maltodextrin and fructose with or without the addition of sodium alginate and pectin and found no differences in ExCHO oxidation between the two conditions, during 120 min of prolonged running at 60% VO_2_max (20). The results of this study are largely in agreement with these studies, showing similar metabolic responses for CHO beverages with or without sodium alginate and pectin. In this study, however, it was also found that serum NEFA concentration was significantly higher throughout the exercise period with the ingestion of ENCAP. That said, there were no other differences between ENCAP and NORM, suggesting that the metabolic response to an isocaloric CHO beverage with or without sodium alginate and pectin is largely similar. However, in contrast to this study and growing literature, there has been a recent publication demonstrating a positive effect of sodium alginate and pectin when ingesting a glucose and fructose beverage at a rate of 90 g^.^h^−1^. This research showed a higher ExCHO oxidation rate, reduced GID symptoms, and improved 5-km time trial when compared to an isocaloric CHO beverage (23). The mechanism through which ExCHO was enhanced with the ingestion of sodium alginate and pectin could not be ascertained by the authors other than potential influences of higher relative exercise intensity and greater enrichment of CHO beverages, increasing the signal-to-noise ratio during the ^13^CO_2_ analysis (23).

A recent review highlighted the lack of evidence for benefits from the use of sodium alginate and pectin added to a CHO beverage on physiological or GID symptoms during moderate intensity exercise (36). Current, mechanism-driven research has shown that a hydrogel can form around a CHO (37), which occurs *in vivo* (18) and subsequently enhances GE (19). Following this, however, there have been several studies which showed no differences from “standard” CHO beverages when comparing various physiological markers (e.g., ExCHO, whole-body oxidation rates and blood metabolites) and subjective markers (e.g., RPE and GID symptoms) [as reviewed elsewhere (36)]. The lack of any clear benefit when ingesting CHO with additional sodium alginate and pectin may be due to several factors. For example, CHO beverages ingested during a GE study will use a single, large bolus of CHO containing a much larger volume of liquid and quantity of CHO than those ingested during studies during exercise (e.g., ~500-mL bolus vs. ~200 mL^.^15 min^−1^). Due to this difference, an effect of the hydrogel is apparent when a large bolus of CHO may disappear when ingesting repeated small volumes during exercise. The lack of any differences in GID during exercise with the addition of sodium alginate and pectin can be attributed to the lack of GID symptoms in the control “standard” CHO beverage. Without GID symptoms in the “control” beverage, it cannot be determined whether the hydrogel exerts a positive effect. A recent study by Rowe et al. (23) has demonstrated that the addition of sodium alginate and pectin is effective at significantly reducing GID symptoms during exercise when GID occurs within the control beverage, suggesting that further investigations with CHO beverages known to cause GID are required. The studies (including this study) comparing GID with an isoenergetic CHO beverage with an hydrogel-based beverage have used relatively low exercise intensities (i.e., ~60–75% VO_2_max) and moderate to high ingestion rates [i.e., 1.3–2.2 g^.^min^−1^, (21, 31, 32)] and thus may not reflect “real-life” conditions during competition, especially at elite level. Although there was also no difference in the GID scores when comparing ENCAP, NORM, and WAT with HiENCAP, three out of seven participants reported at least one symptom as severe with the ingestion of HiENCAP (Table 2). This is, however, unsurprising considering the significant amount of unabsorbed CHO presumed to be present in the intestine with the ingestion of HiENCAP, which is thought to significantly contribute to GID (38). Furthermore, there were no severe GID symptoms reported in the ENCAP or NORM trials and largely similar cumulative scores for GID.

This study has several limitations which must be considered when interpreting the results presented. There was not an isocaloric comparison for HiENCAP which did not contain sodium alginate and pectin, and therefore, we could not establish any potential effects of sodium alginate and pectin at this high ingestion rate, but only confirm if high ExGluc oxidation rates were achievable. Due to a large amount of participant “dropouts,” certain, secondary, outcomes of this study are likely to be under powered, such as blood glucose. In this study, both the maltodextrin and fructose were naturally enriched with ^13^C, with additional ^13^C glucose tracer added to each CHO beverage, to ensure that the expired end-tidal breath collection was sufficiently enriched for measurement. This additional source of ^13^C will introduce an error into the calculation of ExCHO oxidation rate as the tracer will not adequately track the oxidation rate of the combined maltodextrin and fructose. Due to this, ExGluc oxidation was calculated following methods published elsewhere (28). This is an estimation based on theoretical oxidation rates and may not reflect the “true” ExGluc oxidation rate precisely, and therefore, caution should be made when interpreting these data in comparison with other literature. It is worth noting that this error will be consistent in all trials within this study, and thus, comparisons between NORM and ENCAP remain valid.

Due to the widespread, public use of CHO products containing sodium alginate and pectin, a qualitative comparison of GID symptoms in athletes using CHO with or without sodium alginate and pectin during “real-life” competitions (i.e., mass start events) should be conducted. Additionally, further laboratory-based studies comparing the metabolic and gastrointestinal response to ingesting a highly concentrated CHO beverage with sodium alginate and pectin at high exercise intensities (i.e., >80% VO_2_max) should be performed to elucidate any quantifiable ergogenic effects and mechanism.

## Conclusion

In summary, the addition of sodium alginate and pectin to a CHO beverage did not alter the total CHO or ExGluc oxidation rate or fat oxidation rate during prolonged endurance exercise when compared to an isocaloric CHO beverage. Additionally, there appears to be no effect of sodium alginate on GID when ingesting CHO at moderate rates (i.e., 70 g.h^−1^); however, the lack of severe GID symptoms in the isocaloric controls and low statistical power prevents firm conclusions being drawn. A high rate of ExGluc oxidation (i.e., 1.13 g^.^min^−1^) was achieved with a high ingestion rate of maltodextrin and fructose (i.e., 3 g^.^min^−1^). Considering the current disparity between the anecdotal evidence of attenuated GID during “real-life” events and the empirical evidence in a laboratory setting, future studies should investigate and quantify the effect of sodium alginate and pectin on GID during competition and the associated very high exercise intensities (i.e., >80% VO_2_max).

## Data Availability Statement

The raw data supporting the conclusions of this article will be made available by the authors, without undue reservation.

## Ethics Statement

The studies involving human participants were reviewed and approved by University of Stirling Ethics Committee. The patients/participants provided their written informed consent to participate in this study.

## Author Contributions

SS, SG, AB, and YP designed the experiment. SS, SG, and BM-P conducted the data collection and analysis. SS drafted the manuscript, with critical appraisal by all authors. All authors approved the final draft prior to submission.

## Funding

This study was supported by a grant from Maurten AB and the Sub2 Foundation. All drinks were provided by Maurten AB. The authors declare that this study received funding from Maurten AB. The funder was not involved in the study design, collection, analysis, interpretation of data, the writing of this article or the decision where to submit it for publication.

## Conflict of Interest

YP is the founding member of the Sub2 project (www.sub2hrs.com); the Sub2 project is affiliated to a non-trading company (Athlome Limited, UK) that is minor (<1.1%) shareholder of Maurten AB. SS is a Ph.D. student funded partly by the Sub2 project and Maurten AB. SS and SG have received one travel grant each. This research is funded partly from a research grant from the Sub2 project and Maurten AB. None of the authors are paid consultants or have ownership of any patents linked to the present research. The remaining authors declare that the research was conducted in the absence of any commercial or financial relationships that could be construed as a potential conflict of interest.

## Publisher's Note

All claims expressed in this article are solely those of the authors and do not necessarily represent those of their affiliated organizations, or those of the publisher, the editors and the reviewers. Any product that may be evaluated in this article, or claim that may be made by its manufacturer, is not guaranteed or endorsed by the publisher.
